# C-EMDNet: A Nonlinear Morphological Deep Framework for Robust Speech Enhancement

**DOI:** 10.3390/s26061917

**Published:** 2026-03-18

**Authors:** Kais Khaldi, Sahar Almenwer, Afrah Alanazi, Inam Alanazi, Anis Mohamed

**Affiliations:** 1 Department of Computer Science, College of Computer and Information Sciences, Jouf University, Sakaka 72388, Saudi Arabia; smalmenwer@ju.edu.sa; 2 Department of Information System, College of Computer and Information Sciences, Jouf University, Sakaka 72388, Saudi Arabia; aoalenzy@ju.edu.sa (A.A.); aaeniz@ju.edu.sa (I.A.); 3 Department of Mathematics, College of Science, Jouf University, Sakaka 72388, Saudi Arabia; amohmed@ju.edu.sa

**Keywords:** speech enhancement, CEEMDAN, empirical mode decomposition, deep learning, U-Net, nonlinear signal processing, morphological analysis

## Abstract

This study introduces C-EMDNet, a nonlinear speech denoising approach that combines the adaptive decomposition capabilities of Complete Ensemble Empirical Mode Decomposition with Adaptive Noise (CEEMDAN) and a deep convolutional architecture operating directly in the time-intrinsic mode function (IMF) domain. Unlike conventional enhancement methods that rely on fixed time–frequency representations, such as the short-time Fourier transform (STFT), the proposed approach interprets CEEMDAN IMFs as a morphological latent space that captures the multi-scale structure of speech. A U-Net-like network was trained to estimate mode-wise masks, enabling selective noise suppression while preserving the harmonic and formant structures. Experiments on standard noisy speech datasets show that C-EMDNet outperforms classical denoising algorithms and competitive deep learning baselines. These results highlight the promise of nonlinear morphological representations for an alternative framework speech enhancement.

## 1. Introduction

Speech signals are inherently nonlinear, non-stationary, and multi-scale [1,2], exhibiting rapid temporal fluctuations, quasi-periodic excitation patterns and complex interactions between excitation sources and vocal-tract resonances [3]. In real-world acoustic environments, speech is frequently corrupted by additive noise, reverberation, and competing sources, leading to substantial degradation in intelligibility and perceptual quality [4]. Consequently, speech enhancement remains a fundamental challenge in audio signal processing, with critical implications for telecommunications, hearing-assistive devices, robust automatic speech recognition [5] and human–machine interaction.

Early speech enhancement approaches relied on linear filtering and statistical estimations. Classical methods such as spectral subtraction [6], Wiener filtering [3] and the Ephraim–Malah MMSE estimators [7,8] assume stationary noise or Gaussian priors. Although computationally efficient, these techniques often introduce artifacts, such as musical noise [9], and degrade significantly under non-stationary conditions. Subsequent refinements incorporated perceptual models [10], Kalman filtering [11] and Bayesian estimators [12] yet the fundamental limitations imposed by linearity and fixed statistical assumptions remained.

The advent of deep learning has transformed this field. Feedforward DNNs [13], convolutional architectures [14], recurrent networks [15] and attention-based models [16] have demonstrated substantial gains by learning highly nonlinear mappings from noisy to clean speech signals. Time–frequency U-Nets [17], mask-based estimators [12] and time-domain models such as Conv-TasNet [18] and Demucs [19] further advanced performance. Recent developments include transformer-based architectures [20], complex domain networks [21], GAN-based systems [22] and real-time enhancement frameworks [23]. Despite their success, most deep models rely on fixed representations, typically the short-time Fourier transform (STFT) [3] or learned convolutional encoders that do not adapt to the intrinsic, multi-scale and nonlinear structure of speech. This mismatch limits their robustness in highly non-stationary environments, where speech and noise exhibit overlapping rapidly varying spectral patterns.

Given the increasing demand for robust speech enhancement in highly dynamic acoustic environments, nonlinear and morphology-aware representations such as Empirical Mode Decomposition (EMD) [1] and its extensions have become particularly relevant for next generation speech processing systems. In parallel, these have emerged as powerful tools for analyzing nonlinear and non-stationary signals. EMD decomposes a signal into intrinsic mode functions (IMFs) that capture oscillatory modes at various scales. Ensemble EMD (EEMD) [24] and Complete Ensemble EMD with Adaptive Noise (CEEMDAN) [25] improved robustness, reduced mode mixing and ensured better IMF alignment across signals. These methods have been successfully applied in biomedical engineering, geophysics and mechanical diagnostics [26]. In speech processing, EMD-based techniques have been explored for pitch estimation [27], feature extraction [28] and noise reduction [29,30,31]. However, prior work has predominantly used EMD as a preprocessing step or handcrafted filter rather than as a learnable representation integrated within a deep neural architecture.

A key limitation of existing research is the absence of a unified framework that combines the adaptive nonlinear decomposition of CEEMDAN with the representational power of deep learning. Previous studies typically apply EMD as a standalone denoising stage or treat IMFs as fixed features [27], without exploiting the potential of the time–IMF domain as a structured, morphology-aware latent space. However CEEMDAN offers the properties of adaptivity, multi-scale resolution, noise robustness and physical interpretability, which make it an ideal foundation for constructing a nonlinear representation tailored to speech enhancement [24,25].

In this study, we introduce C-EMDNet, a nonlinear morphological deep framework for robust speech enhancement. Building upon the adaptive decomposition capabilities of CEEMDAN and the representational power of convolutional neural networks, the proposed approach departs from conventional STFT-based pipelines and operates directly in the time–IMF domain.

**Figure 1:**
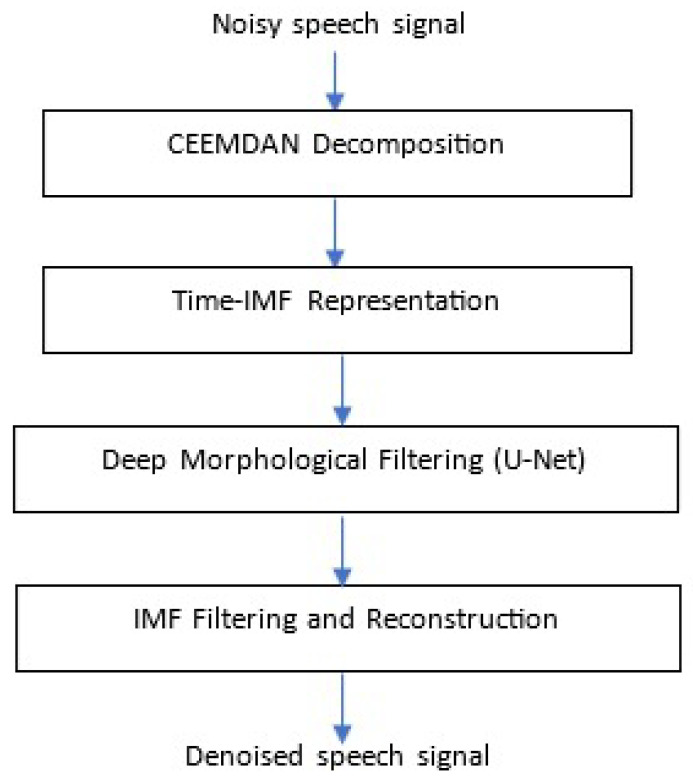
Overview of the proposed approach C-EMDNet.

Figure 1 provides an overview of the proposed C-EMDNet framework. The model combines CEEMDAN-based adaptive decomposition with a deep U-Net operating in the time–IMF domain, enabling nonlinear, morphology-aware filtering. The main contributions of this study are summarized as follows:

1. **A novel morphological representation for speech enhancement.** We propose an approach that treats CEEMDAN intrinsic mode functions (IMFs) as a learnable morphological latent space. Unlike fixed time–frequency representations, such as the STFT, CEEMDAN provides an adaptive, nonlinear and multi-scale decomposition aligned with the intrinsic dynamics of speech [1,24,25]. This enables a representation that is inherently robust to non-stationary noise.
2. **A deep U-Net architecture operating directly in the time–IMF domain.** We design a U-Net-like network [17] that performs convolution across IMFs and time, learning mode-wise masks that selectively suppress noise-dominated components while preserving harmonic and formant structures. This constitutes the integration of CEEMDAN and deep learning in a unified, fully trainable framework.
3. **A principled fusion of nonlinear decomposition and data-driven learning.** C-EMDNet leverages the complementary strengths of CEEMDAN and neural networks: CEEMDAN provides interpretable morphological cues and oscillatory modes, whereas the network learns optimal filtering strategies within this adaptive morphological space. This overcomes the limitations of prior EMD-based methods that relied on handcrafted rules [29,30,31] or were used only as preprocessing steps [27].
4. **Enhanced robustness to non-stationary and real-world noise.** By operating on IMFs that naturally separate the speech structure from noise, C-EMDNet achieves fine-grained denoising and improved perceptual quality. The model demonstrates strong robustness across diverse noise types and SNR conditions, outperforming classical algorithms [3,6,7] and recent deep learning baselines, including GAN-based [32], transformer-based [16] and complex-domain models [33].

The main methodological novelty of C-EMDNet lies in the introduction of a nonlinear, morphology-aware latent space derived from CEEMDAN, combined with a deep U-Net that performs convolution directly across IMFs and time. This design differs fundamentally from waveform-based and vocoder-based approaches, which rely on fixed or learned representations that do not explicitly capture the intrinsic oscillatory structure of speech. To evaluate C-EMDNet, we conduct extensive experiments on the VoiceBank-DEMAND dataset [34] across diverse noise types and SNR conditions. Results show that C-EMDNet consistently outperforms classical denoising algorithms [3,6,7,8] and competitive deep learning baselines [16,32,35,36], including recent transformer and GAN-based models [33,37]. These findings demonstrate the potential of nonlinear morphological representations for next-generation speech enhancement.

The remainder of this paper is organized as follows. Section 2 presents a literature review on speech enhancement, deep learning architectures and EMD-based signal analysis. Section 3 explores the theoretical background of EMD and its extensions. Section 4 presents the proposed approach C-EMDNet, detailing the CEEMDAN-based front-end and mode-dependent deep filtering architecture. Section 5 describes the experimental setup, datasets, noise conditions and evaluation metrics. Section 6 presents the quantitative and qualitative results, including comparisons with classical, deep learning and recent state-of-the-art approaches. Finally, Section 7 concludes the paper and outlines the recommended research directions.

## 2. Related Work

Speech enhancement has been investigated using several methodological paradigms, including classical signal processing, deep learning and adaptive nonlinear decomposition. This section reviews these research directions and highlights the limitations that motivate the development of the proposed approach C-EMDNet.

### 2.1. Classical Speech Enhancement

Classical enhancement techniques rely on linear filtering and statistical modeling. Spectral subtraction [6], Wiener filtering [3] and MMSE estimators, such as the Ephraim-Malah algorithms [7,8], assume stationary noise or Gaussian priors. Although computationally efficient, these methods often introduce musical noise artifacts [9] and degrade significantly in non-stationary environments. Extensions incorporating perceptual weighting [10], Kalman filtering [11] and Bayesian estimators [12] improved robustness but remained constrained by the linearity and fixed statistical assumptions inherent to their formulations, respectively. Consequently, classical approaches struggle to model the nonlinear, multi-scale, and rapidly varying structures of real-world speech signals.

### 2.2. Deep Learning-Based Speech Enhancement

Deep learning has become the dominant paradigm for speech enhancement because of its ability to learn highly nonlinear mappings from noisy to clean speech. Early feedforward DNNs [13], CNN-based architectures [14], and LSTM models [15] have demonstrated substantial improvements over classical methods. Time–frequency U-Nets [17], mask-based estimators [12] and time-domain models such as Conv-TasNet [18], Wave-U-Net [14] and Demucs [19] further advanced performance by leveraging hierarchical feature extraction and end-to-end waveform modeling.

Recent developments include transformer-based architectures [20], complex-domain networks such as Deep Complex U-Net [21], and phase-aware models like PHASEN and PHASEN++ [16,38]. GAN-based systems, including SEGAN [35], MetricGAN [32] and MetricGAN+ [22], introduced perceptually motivated objectives that correlate better with human auditory judgments. Recently, real-time enhancement frameworks [23] and comparative evaluations of modern deep models [39] have highlighted the increasing maturity and diversity of data-driven approaches.

Despite these advances, most deep learning systems rely on fixed representations, typically the short-time Fourier transform (STFT) [3] or learned convolutional encoders that do not adapt to the intrinsic, nonlinear and multi-scale structure of speech. Fixed-resolution transforms struggle to capture rapid transitions, harmonic noise interactions and non-stationary fluctuations, limiting the robustness of deep models under challenging acoustic conditions. These limitations motivate the exploration of adaptive morphology-aware representations.

Recent studies have also explored waveform-level acoustic modeling and vocoder refinement strategies. For example, Al-Radhi et al. [40] investigated noise-aware waveform generation and spectral modeling for text-to-speech and speech conversion tasks, highlighting the importance of robust acoustic representations in noisy conditions. While such approaches rely on learned waveform generators or vocoder structures, the proposed C-EMDNet differs fundamentally by introducing a nonlinear, morphology-aware latent space derived from CEEMDAN. This enables mode-wise filtering of intrinsic oscillatory components, providing a complementary alternative to both waveform-based and vocoder-based modeling strategies.

Additional refinements in pitch tracking and Harmonic to Noise Ratio (HNR) estimation have been explored within vocoder frameworks. For example, Al Radhi et al. [40] proposed adaptive improvements to pitch modeling and HNR estimation for statistical parametric speech synthesis. While such methods rely on parametric representations of excitation and spectral envelope, the proposed C-EMDNet differs fundamentally by operating in a nonlinear, morphology-aware domain derived from CEEMDAN. This allows pitch-related oscillations, harmonic structures, and noise components to be separated naturally across IMFs, enabling mode-wise filtering without explicit parametric modeling. As a result, C-EMDNet provides a complementary alternative to vocoder-based refinements, particularly under non-stationary noise conditions.

### 2.3. EMD-Based Speech Processing

Empirical Mode Decomposition (EMD) and its extensions have been applied to speech processing for pitch estimation [27], feature extraction [28], voiced/unvoiced classification [31] and noise reduction [29,30]. These methods exploit the ability of the EMD to decompose speech into intrinsic mode functions (IMFs) that capture oscillatory patterns at different scales. However, existing EMD-based approaches typically rely on handcrafted rules, IMF selection heuristics and fixed filtering strategies. They operate as standalone signal processing tools and lack the adaptability, optimization capability and representational power of contemporary deep learning architectures. Importantly, prior studies did not integrate EMD or CEEMDAN into fully trainable unified framework neural networks.

## 3. Background

This section provides the theoretical foundations necessary to understand the proposed approach C-EMDNet. We review Empirical Mode Decomposition (EMD) and its extensions and outline the principles of deep learning-based speech enhancement without discussing prior works or their limitations.

### 3.1. Empirical Mode Decomposition and CEEMDAN

Empirical Mode Decomposition (EMD), introduced by Huang et al. [1], is a fully data-driven method for analyzing nonlinear and non-stationary signals. Unlike Fourier or wavelet transforms [41], the EMD does not rely on predefined basis functions. Instead, it decomposes a signal into intrinsic mode functions (IMFs), each representing an oscillatory component with a well-defined instantaneous frequency. The decomposition is obtained through an iterative sifting process that enforces local symmetry and ensures that each IMF satisfies specific oscillatory constraints.

Ensemble EMD (EEMD) [24] improves robustness by adding white noise and averaging multiple decompositions; however it does not guarantee perfect reconstruction. The complete Ensemble EMD with Adaptive Noise (CEEMDAN), introduced by Torres et al. [25], addresses these limitations by ensuring complete reconstruction, reducing mode mixing and improving IMF alignment across signals. These properties make CEEMDAN particularly suitable for constructing adaptive multi-scale representations of speech [2,42].

### 3.2. EMD-Like Methods in Speech Processing

EMD-based methods have been used in speech processing for tasks such as pitch estimation [27], feature extraction [28] and noise reduction [29,30,31]. These applications demonstrate that the IMFs capture meaningful oscillatory structures in speech. However, these methods typically operate as standalone signal-processing tools and are not integrated into end-to-end learning frameworks.

### 3.3. Deep Learning Principles for Speech Enhancement

Deep learning-based speech enhancement methods operate either in the time–frequency domain or directly in the time domain. Time–frequency approaches use the STFT to obtain magnitude or complex spectra and apply neural networks for spectral mapping or mask estimation [13,16,17]. Time-domain models, such as Conv-TasNet [18], Demucs [19] and Wave-U-Net [14], learn end-to-end mappings from noisy to clean waveforms using convolutional encoders and decoders.

GAN-based models [32,35] introduce adversarial or perceptual objectives to improve perceptual quality of the generated audio. These architectures provide strong denoising performance but rely on fixed representations that do not adapt to the intrinsic speech structure.

## 4. Proposed Method

CEEMDAN provides a decomposition that is nonlinear, adaptive, multi-scale and robust to noise [24,25]. These properties make the time–IMF domain an attractive candidate for constructing a morphological latent space for speech enhancement. The proposed approach C-EMDNet leverages this decomposition by treating IMFs as structured, interpretable features, learning mode-wise masks through a U-Net architecture [17], reconstructing speech from filtered IMFs and exploiting intrinsic oscillatory structures that fixed transforms cannot capture. This theoretical foundation supports the development of a new class of nonlinear morphological deep learning models for speech enhancement.

This section presents the full processing pipeline, from the morphological front-end to the reconstruction and learning objectives, and highlights how each component contributes to robust and structure-aware denoising.

### 4.1. Overview of the C-EMDNet Architecture

Unlike STFT-based systems that rely on a fixed time–frequency grid and assume local stationarity [3,6], C-EMDNet operates in a nonlinear, adaptive morphological domain derived from CEEMDAN [24,25]. This representation aligns naturally with the intrinsic oscillatory structure of speech and enables mode-wise filtering strategies that are not accessible through conventional transforms.

The processing pipeline consists of four stages:

1. CEEMDAN decomposition of the noisy speech signal;
2. Construction of a multichannel time–IMF tensor;
3. Deep morphological filtering via a 2D U-Net;
4. Reconstruction of the enhanced speech signal from filtered IMFs.

This design explicitly exploits the multi-scale, nonlinear structure of speech [1,43], enabling fine-grained discrimination between speech-dominant and noise-dominant oscillatory modes.

### 4.2. CEEMDAN as a Nonlinear Morphological Front-End

Given a noisy speech signal x(t), CEEMDAN produces the following decomposition:

(1) $$x\left(t\right)=\sum_{k=1}^{K}c_{k}\left(t\right)+r\left(t\right),$$

where $c_{k}\left(t\right)$ denotes the *k*-th intrinsic mode function (IMF), ordered from high to low oscillatory content, and r(t) denotes the residual trend.

CEEMDAN improves upon classical EMD by:

- Injecting adaptive noise at each iteration;
- Ensuring complete and stable reconstruction;
- Reducing mode mixing;
- Improving IMF alignment across signals [25,42].

These properties are essential for supervised learning because they guarantee consistent IMF indexing across training examples and preserve meaningful oscillatory structures. In practice, CEEMDAN naturally separates:

- High-frequency IMFs dominated by noise;
- Mid-frequency IMFs containing harmonics and formants;
- Low-frequency IMFs encoding prosodic contours.

This makes CEEMDAN an ideal nonlinear front-end for speech enhancement, providing a morphology-aware decomposition that complements deep neural filtering.

### 4.3. Construction of the Time–IMF Tensor

The extracted IMFs are arranged in a time-mode matrix as follows:

(2) $$C\left(t,k\right)=c_{k}\left(t\right),\;t=1,\ldots ,T,\;k=1,\ldots ,K.$$

To enrich this representation, additional descriptors were computed for each IMF using the Hilbert transform:

(3) $$\begin{array}{cc} \;a_{k}\left(t\right) & =|H\{c_{k}\left(t\right)\}|, \end{array}$$

(4) $$\begin{array}{cc} \;\omega_{k}\left(t\right) & =\frac{d}{dt}\arg\left(H\{c_{k}\left(t\right)\}\right), \end{array}$$

(5) $$\begin{array}{cc} e_{k}\left(t\right) & =c_{k}^{2}\left(t\right), \end{array}$$

where H{·} denotes the Hilbert operator.

These descriptors capture the instantaneous amplitude, frequency and energy variations, providing complementary morphological cues. They were concatenated into a 3D tensor as follows:

(6) $$F\left(t,k,d\right)\in R^{T\times K\times D},$$

where *D* is the number of feature channels (typically D=4: raw IMF, envelope, instantaneous frequency, and energy). To mitigate known limitations of Hilbert-based analysis, specifically boundary effects and negative-frequency instabilities, we applied several stabilization steps during the computation of instantaneous frequency. First, CEEMDAN produces IMFs with improved local symmetry and reduced mode mixing, which naturally alleviates edge distortions in the Hilbert transform. Second, each IMF was symmetrically extended prior to Hilbert processing to suppress boundary-induced artifacts. Third, occasional negative instantaneous frequencies, which arise from numerical instabilities and do not correspond to providing interpretable morphological cues oscillations, were clipped to zero following standard EMD–Hilbert practice. Finally, because the U-Net operates on the complete time–IMF tensor, the network implicitly learns to down-weight unreliable regions near boundaries. These steps ensure that the instantaneous frequency descriptor remains stable and informative for morphological learning. This tensor forms a nonlinear, multi-scale, morphology-aware latent space that is significantly more expressive than fixed spectral representations [41,43]. This enables the network to jointly exploit the temporal dynamics and intermode relationships.

### 4.4. Deep Morphological Filtering via U-Net

The tensor F(t,k,d) provides a structured three-dimensional representation that jointly encodes temporal evolution, the hierarchy of intrinsic mode functions (IMFs) and their associated morphological descriptors. To exploit this rich organization, we employ a 2D U-Net architecture [17], which is particularly well-suited for learning interactions across time and IMF dimensions.

In this formulation, the horizontal axis corresponds to time, the vertical axis to the ordered set of IMFs and the channel dimension to the feature descriptors (raw IMF, amplitude envelope, instantaneous frequency and instantaneous energy). This arrangement enables the network to simultaneously capture: (i) local temporal patterns within each IMF, (ii) cross-mode dependencies reflecting coherent oscillatory activity across IMFs and (iii) multi-scale structures characteristic of non-stationary signals.

The encoder progressively aggregates information by reducing the spatio modal resolution, thereby extracting increasingly abstract and hierarchical representations. The decoder then reconstructs fine-grained details through upsampling operations and skip connections, which reintroduce high-resolution features that were lost during encoding. This combination of global contextual modeling and local detail preservation is particularly advantageous in the time–IMF domain, where oscillatory modes exhibit structured and non-stationary correlations.

The network outputs a mode-wise mask

(7) M(t,k)∈[0, 1],

which modulates each IMF according to

(8) $${\hat{c}}_{k}\left(t\right)=M\left(t,k\right)\;c_{k}\left(t\right).$$

Unlike STFT-based masking, which operates on fixed frequency bins, this approach filters intrinsic oscillatory modes, enabling more precise discrimination between speech and noise while mitigating phase-related artifacts commonly observed in spectral-masking methods. Finally, the estimated mask M(t,k) is implicitly shaped by the combined loss functions for morphological learning, which guides the U-Net to suppress noise, preserve speech-dominant oscillations, reconstruct a clean waveform, maintain perceptual naturalness and respect the intrinsic morphology of the IMFs.

#### 4.4.1. Loss Functions for Morphological Learning

To train the proposed model, we employ three complementary loss functions that jointly constrain the network to produce enhanced speech that is temporally faithful, perceptually natural and morphologically consistent with the intrinsic oscillatory structure of the clean IMFs. Each loss targets a distinct aspect of the reconstruction process and their combination provides a balanced supervision signal to learn an effective mode-wise mask.

##### Domain Reconstruction Loss

The objective operates directly in the waveform domain and penalizes sample-wise deviations between the enhanced signal $\hat{s}\left(t\right)$ and the clean reference s(t):

(9) $$L_{\mathrm{time}}={∥\hat{s}\left(t\right)-s\left(t\right)∥}_{1}.$$

The $L_{1}$ norm is chosen for its robustness to outliers and its ability to preserve sharp temporal structures, thereby encouraging accurate waveform reconstruction.

##### Perceptual Loss

While the time-domain loss enforces sample level fidelity, it does not fully capture perceptual attributes that correlate with human auditory perception. To address this limitation, we incorporate a perceptual loss defined as

(10) $$L_{\mathrm{perc}}={∥\Phi \left(\hat{s}\right)-\Phi \left(s\right)∥}_{2}^{2},$$

where Φ(·) denotes a pretrained perceptual feature extractor, such as MetricGAN [32]. This loss encourages the enhanced signal to match the clean reference in a perceptually meaningful feature space, promoting naturalness and reducing artifacts that may not be captured using purely time-domain criteria.

##### Morphological Consistency Loss

Given that the proposed approach operates in the time–IMF domain. The intrinsic morphology of speech-dominant IMFs is essential for understanding their characteristics. To this end, we introduce a morphological consistency loss that penalizes deviations between the filtered IMFs ${\hat{c}}_{k}\left(t\right)$ and their clean counterparts $c_{k}^{\mathrm{clean}}\left(t\right)$:

(11) $$L_{\mathrm{morph}}=\sum_{k=1}^{K}{∥{\hat{c}}_{k}\left(t\right)-c_{k}^{\mathrm{clean}}\left(t\right)∥}_{1}.$$

This mechanism prevents over-suppression of speech-relevant oscillations and ensures that the oscillatory patterns characteristic of clean speech are preserved across all modes.

##### Final Objective

The overall training objective is a weighted combination of the three losses:

(12) $$L=\alpha L_{\mathrm{time}}+\beta L_{\mathrm{perc}}+\gamma L_{\mathrm{morph}},$$

where the coefficients (α,β,γ) control the relative importance temporal accuracy, perceptual quality and morphological preservation. The weighting coefficients in Equation (12) were tuned using a restricted grid-search procedure. Candidate values for ($L_{\mathrm{time}}$, $L_{\mathrm{perc}}$, $L_{\mathrm{morph}}$) were evaluated on a held-out validation subset, and the optimal triplet was selected using a multi-objective criterion that jointly maximized PESQ, STOI, SI-SDR, and IMF-wise stability. This approach ensures a balanced trade-off between perceptual quality, temporal fidelity, and morphological consistency. A sensitivity analysis further confirmed that the model remains stable across a broad range of hyperparameter values.

The resulting mask M(t,k) is therefore implicitly shaped by this composite loss, guiding the U-Net to suppress noise, retain speech-dominant oscillations, reconstruct a clean waveform and maintain the intrinsic structure of the IMFs.

#### 4.4.2. Mask Determination Factors

The estimated mask M(t,k) is not determined by a single component of the framework, but rather emerges from the interaction of several complementary factors that jointly guide U-Net during training. These factors shape how the network distinguishes speech-dominant oscillations from noise across the IMF hierarchy.

##### Input IMFs and Morphological Descriptors

The primary driver of the mask is the structure of the noisy IMFs $\left\{c_{k}\left(t\right)\right\}$ and their associated morphological descriptors F(t,k,d). These descriptors including the raw IMF, amplitude envelope, instantaneous frequency and instantaneous energy provide the network with rich information on the local oscillatory behavior of each mode. As a result, M(t,k) adapts to the time-varying morphology of the input signal.

##### Network Architecture

The 2D U-Net plays a central role in determining the masks. Its convolutional layers, multi-scale receptive fields and skip connections enable the model to capture both local temporal patterns and cross-mode dependencies. The architecture therefore learns how to combine morphological features across time and IMFs to produce a coherent mode-wise mask.

##### Training Losses

The three loss functions introduced earlier provide complementary supervision that shape the behavior of the mask. The time-domain loss enforces waveform fidelity, the perceptual loss encourages natural sounding reconstruction and the morphological consistency loss preserves the intrinsic oscillatory structure of the speech. Together, these losses constrain the mask to suppress noise while retaining speech-relevant oscillations.

##### Loss Weights

The coefficients (α,β,γ) control the relative influence of three losses during the optimization. Although they do not directly define the mask, they regulate the balance between temporal accuracy, perceptual quality and morphological preservation. Appropriate weighting ensures that the learned mask achieves effective noise suppression without degrading the structure of speech-dominant IMFs.

##### Overall Effect

In summary, the mask M(t,k) results from the combined effect of the input IMFs, their morphological descriptors, the U-Net architecture and multi-objective loss function. This interplay enables the model to produce a mask that suppresses noise, preserves useful oscillations, reconstructs a clean waveform and maintains the intrinsic morphology of the IMFs.

### 4.5. Reconstruction of the Enhanced Speech Signal

The enhanced speech signal is reconstructed by summing the filtered IMFs as follows:

(13) $$\hat{x}\left(t\right)=\sum_{k=1}^{K}{\hat{c}}_{k}\left(t\right).$$

This reconstruction preserves the morphological structure of speech and avoids the phase reconstruction issues and musical noise artifacts commonly observed in STFT-based methods [7,9]. Because CEEMDAN ensures complete reconstruction, the enhanced signal remains faithful to the underlying oscillatory pattern.

## 5. Experimental Setup

### 5.1. Dataset

All speech material used in this study was exclusively drawn from the VoiceBank-DEMAND corpus [34], which contains 11,572 utterances produced by multiple speakers and mixed with real environmental noise from the DEMAND database. To structure the acoustic variability of the dataset, the DEMAND noise recordings were reorganized into four categories according to their temporal characteristics: stationary noise (e.g., ventilation, steady engine hum), quasi-stationary noise (e.g., continuous traffic flow, cafeteria ambience), non-stationary noise (e.g., metro announcements, door slams, human activity) and natural environmental noise (e.g., park recordings, outdoor urban scenes) [14,44]. To rigorously evaluate the generalization capability of the proposed architecture C-EMDNet, we conducted extensive testing on 100 previously unseen speech signals originating from 10 different speakers in the VoiceBank-DEMAND corpus. These test signals were mixed with DEMAND noise at varying SNR levels, enabling a comprehensive comparison between C-EMDNet and several classical speech enhancement approaches. This experimental design ensured that the reported improvements reflected consistent robustness across diverse acoustic environments and speaker variations.

### 5.2. Noise Characteristics and Mixing Conditions

The noisy signals provided by the VoiceBank-DEMAND corpus encompass a broad range of real-world perturbations with varying temporal and spectral characteristics. For clarity, we categorized these noises into four conceptual classes:

**Stationary noises** exhibit stable spectral statistics over time, such as steady appliance hums or ventilation noise. These conditions are useful for analyzing the model behavior under slowly varying or constant noise profiles [6].

**Quasi-stationary noises** include environments such as office chatter or idling engines, where the spectral envelope evolves gradually. These noises test the model’s ability to track moderate temporal variations [7].

**Non-stationary noises** such as traffic, street activity and public spaces contain abrupt spectral changes and overlapping acoustic events. These conditions are particularly challenging for STFT-based systems because of their local stationarity assumptions [3].

**Natural environmental noises** (e.g., wind, rain, outdoor ambience) introduce broadband, nonlinear and rapidly fluctuating structures [2], making them ideal for evaluating the benefits of CEEMDAN adaptive decomposition.

The dataset includes mixtures at multiple signal-to-noise ratios (SNRs), typically ranging from 0 dB to 15 dB. Low-SNR conditions are especially important, as deep learning models often experience significant degradation below 0 dB [45].

### 5.3. Preprocessing and CEEMDAN Decomposition

All audio signals were normalized and resampled at 16 kHz. CEEMDAN decomposition was performed following the procedures described in [24,25]. For each utterance, the decomposition produced a set of intrinsic mode functions (IMFs) capturing oscillatory behaviors from high-frequency fricatives to low-frequency prosodic contours [27]. The number of IMFs was selected to ensure coverage of the full speech bandwidth while maintaining a consistent IMF indexing across samples. In practice, CEEMDAN produces a variable number of IMFs depending on the signal complexity. For each noisy–clean pair, we use the number of IMFs generated from the noisy signal as the reference. If the clean signal produces fewer IMFs, additional residual modes are appended through zero-padding so that both decompositions share the same number of IMFs. This alignment ensures that IMF index k corresponds to a comparable oscillatory scale across paired utterances, despite the variable-length nature of CEEMDAN.

CEEMDAN provides a nonlinear, adaptive, and multi-scale representation that separates noise-dominated components from speech-dominant structures more effectively than fixed spectral transforms [1]. This decomposition forms the morphological front-end used by C-EMDNet. Although CEEMDAN follows a fixed algorithmic procedure with predefined stopping criteria and noise injection parameters, the resulting IMFs are adaptive to the input signal because they are derived from its local extrema and intrinsic oscillatory patterns. This contrasts with fixed spectral transforms such as STFT, which rely on predetermined basis functions independent of the waveform.

### 5.4. Training Procedure

C-EMDNet was trained using the Adam optimizer [46] with mini-batches of paired noisy clean utterances randomly sampled from the training set. The learning objective combines three complementary losses: (i) a time-domain reconstruction loss promoting waveform fidelity, (ii) a perceptual loss encouraging naturalness in the enhanced signal and (iii) a morphological consistency loss preserving the oscillatory structure of speech [32].

This multi-objective formulation ensures that the model captures temporal accuracy, perceptual quality and structural coherence simultaneously. The training was performed for a fixed number of epochs with early stopping based on the validation performance.

To assess the stability of the proposed framework, we conducted a sensitivity analysis focusing on the key hyperparameters of the model, including the U-Net depth, channel width, and the weighting coefficients of the different loss terms. Unlike approaches that fix the number of IMFs, our method processes all IMFs generated by CEEMDAN, whose number naturally varies from one signal to another. For this reason, instead of artificially fixing K, we evaluated the effect of selectively removing specific IMF groups (e.g., the highest-frequency IMFs or the lowest-frequency trend-related IMFs). The results show that C-EMDNet maintains stable performance even when certain IMFs are removed, confirming the robustness of the CEEMDAN mode decomposition. Furthermore, variations in U-Net depth, channel width, or loss weighting coefficients lead to only minor fluctuations in PESQ, STOI, and SI-SDR, demonstrating strong generalization and stable training behavior.

The proposed C-EMDNet model was trained for 120 epochs using the Adam optimizer with an initial learning rate of $1\times 10^{-4}$, reduced by a factor of 0.5 every 20 epochs based on validation loss. A batch size of 16 was used throughout training. The CEEMDAN decomposition produced K=19 IMFs, of which the first K=18 were retained for training and inference, as the last mode corresponds to residual low-frequency energy. The U-Net backbone consists of 5 encoder–decoder levels with channel widths {32, 64, 128, 256, 512} and symmetric skip connections. The weighting coefficients (α, β, γ) in the composite loss function were set to (1.0,0.5,2.0), following the restricted grid-search and multi-objective validation strategy described earlier.

### 5.5. Evaluation Metrics

The performance was assessed using three widely adopted metrics:

- PESQ (Perceptual Evaluation of Speech Quality), which correlates with subjective quality judgments;
- STOI (Short-Term Objective Intelligibility), which measures speech intelligibility;
- SI-SDR (Scale-Invariant Signal-to-Distortion Ratio), which quantifies waveform-level reconstruction accuracy.

These metrics are standard in recent enhancement research [13,14,44] and enable direct comparisons with existing baselines.

For each metric (PESQ, STOI, SI-SDR), we report both the mean and the standard deviation computed across all test utterances. Since inference is deterministic and the test set is large, standard deviations provide a direct and meaningful measure of variability, whereas confidence intervals are less informative in this context.

## 6. Results and Discussion

This section provides a comprehensive evaluation of the proposed approach C-EMDNet. The analysis combines qualitative inspection of the waveform and temporal reconstructions across four noise categories, followed by a quantitative comparison using PESQ, STOI and SI-SDR.

### 6.1. Qualitative Analysis and Spectrogram Evaluation

**Figure 2:**
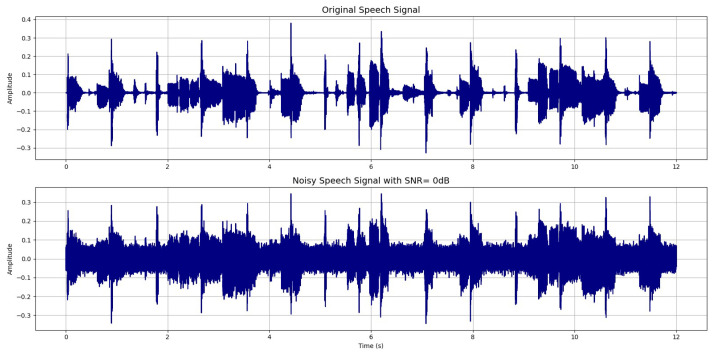
Clean and noisy speech signals (SNR=0 dB).

To evaluate the robustness of the proposed approach under severe noise conditions, the clean signal was artificially contaminated with stationary noise at an SNR of 0 dB. This configuration represents an extremely challenging scenario in which the noise power equals the speech power. Both the clean reference signal and its noisy counterpart are presented in Figure 2, illustrating the significant degradation introduced by the noise.

**Figure 3:**
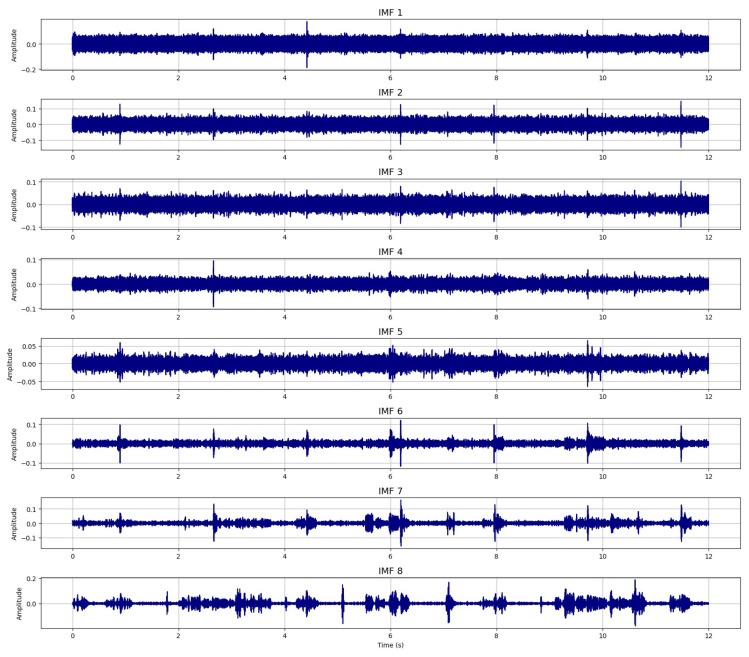
CEEMDAN decomposition of the noisy speech signal (First eight IMFs).

Figure 3 illustrates the CEEMDAN-based decomposition of the noisy speech signal, which has been corrupted by stationary noise at an SNR of 0 dB. The decomposition produces a total of 19 intrinsic mode functions (IMFs), capturing oscillatory components from high to low frequency. For clarity and improved readability, only the first eight IMFs, those containing the most relevant high-frequency structures, are displayed in the figure.

**Figure 4:**
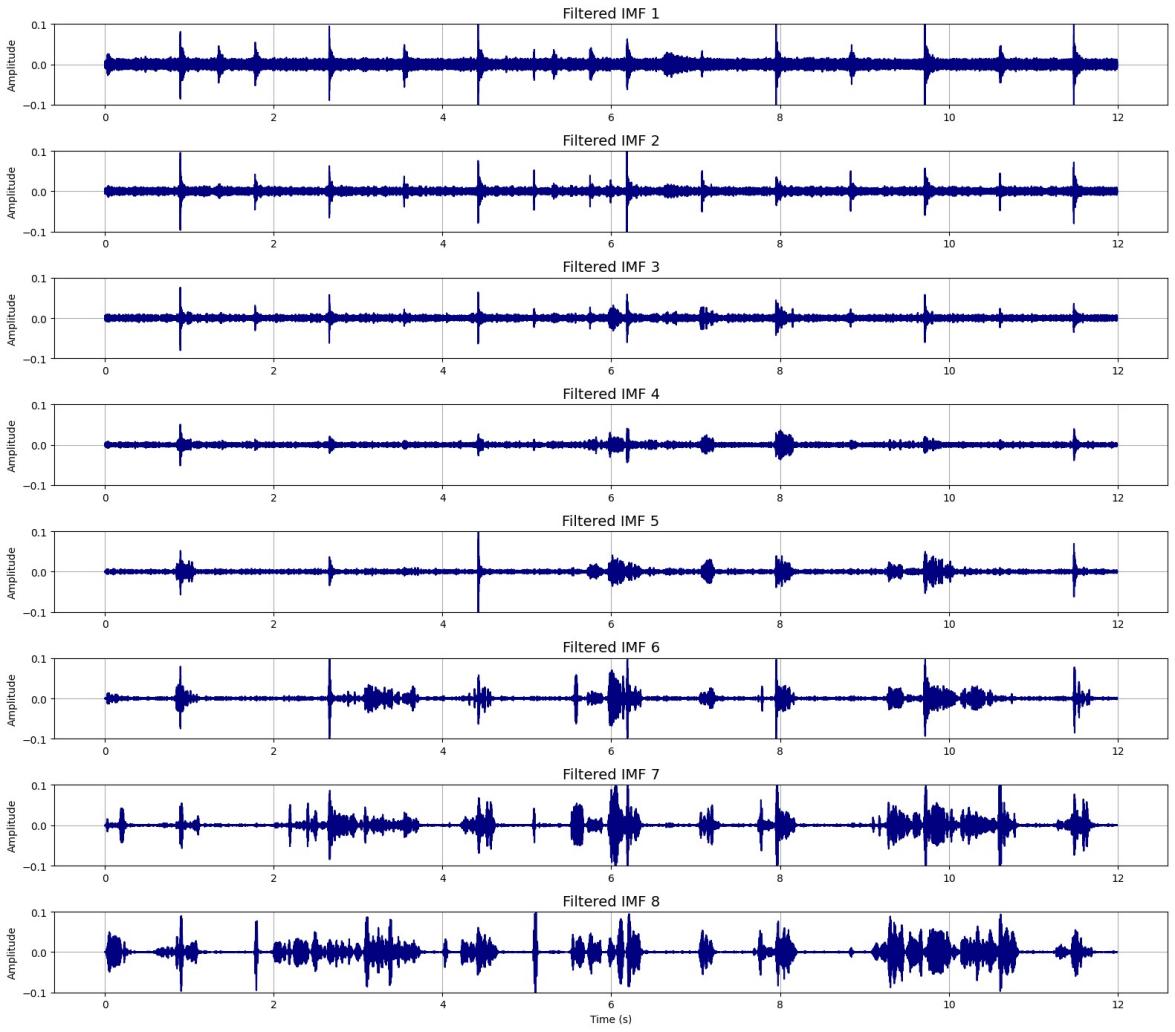
U-Net-filtered IMFs (first eight IMFs) within the proposed approach C-EMDNet.

Within the proposed approach C-EMDNet, each noisy IMF is processed and filtered using a U-Net-based architecture specifically designed to preserve fine-grained temporal structures. Figure 4 presents the resulting filtered IMFs, which exhibit a substantial reduction in noise while maintaining the intrinsic oscillatory patterns of the original components.

**Figure 5:**
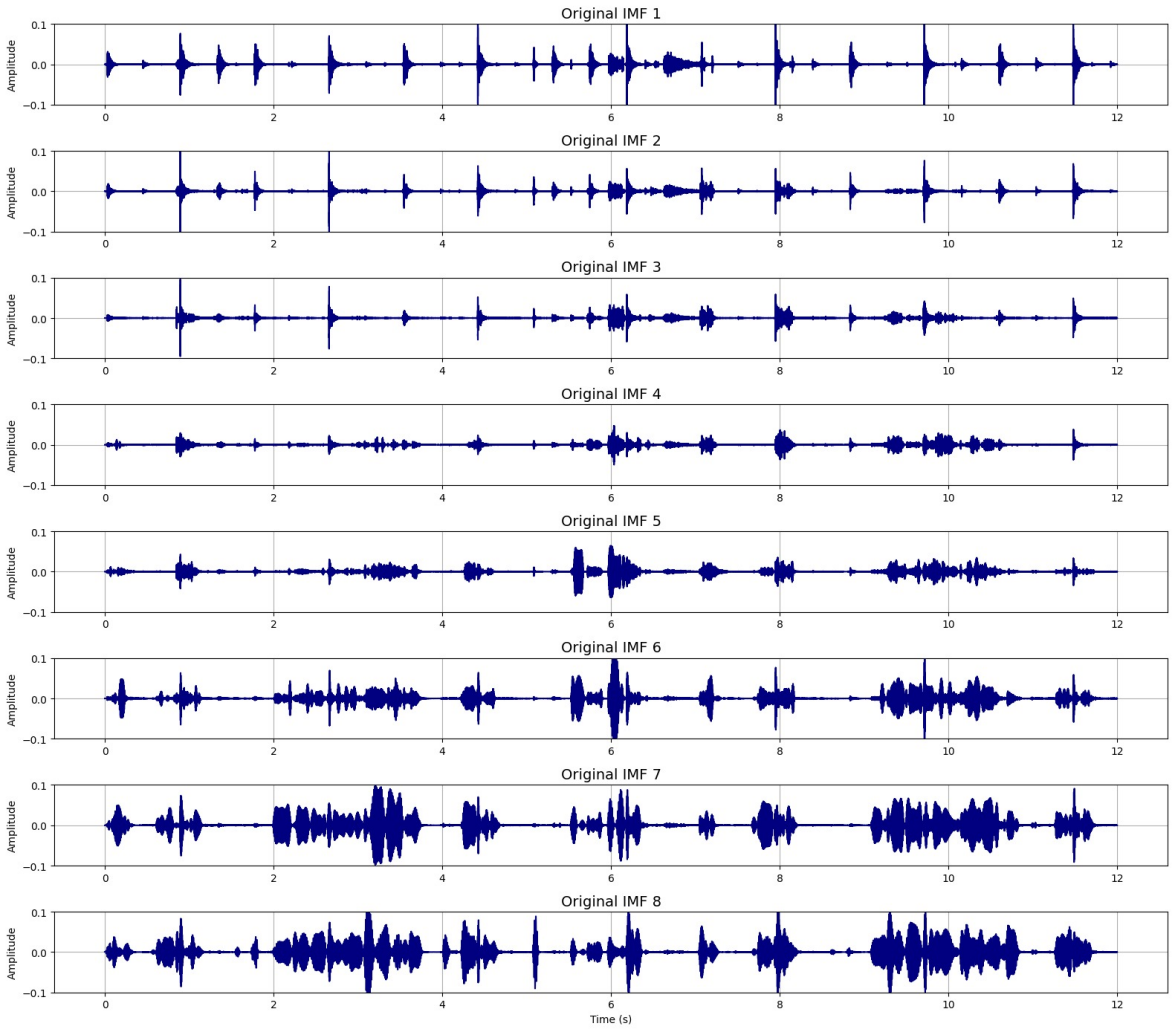
CEEMDAN decomposition of the clean speech signal (first eight IMFs).

By comparing the filtered IMFs with the original IMFs extracted from the clean speech signal in Figure 5, we observe that the filtered components closely follow their clean counterparts, both in amplitude and oscillatory structure.

**Figure 6:**
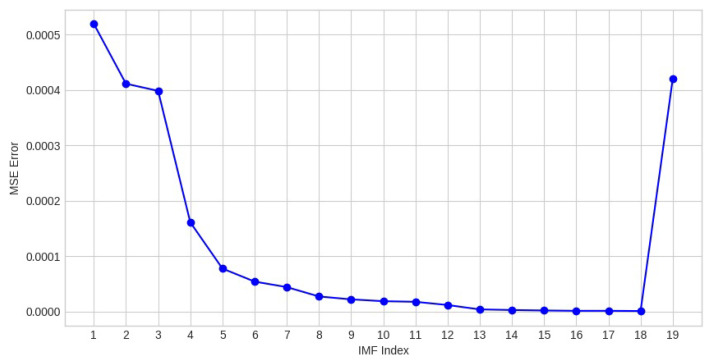
IMF-wise reconstruction error between clean and enhanced speech signals.

Figure 6 reports the Mean Square Error (MSE) between each filtered IMF and its corresponding clean IMF. A detailed analysis reveals that the reconstruction errors are most pronounced in the first high-frequency IMFs. This behavior is expected, as noise is predominantly concentrated in the highest frequency modes, naturally leading to larger deviations in these components. From approximately the fifth IMF onward, the MSE progressively diminishes and approaches zero, reflecting the effectiveness of the deep learning stage in selecting and filtering the most informative IMFs. A noticeable increase in MSE is also observed in the final IMF (IMF19). This outcome is entirely consistent with the CEEMDAN decomposition, which yields only 18 meaningful IMFs for the original speech signal. Consequently, the MSE associated with IMF19 does not represent a genuine reconstruction discrepancy but instead corresponds to the residual average energy of this mode.

**Figure 7:**
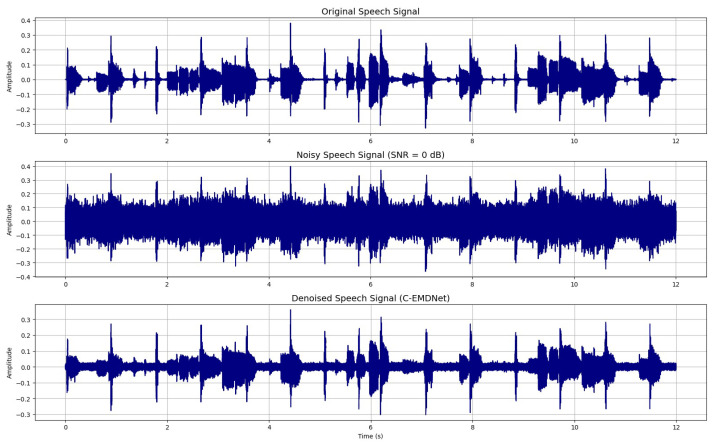
Speech denoising results: original speech signal, noisy speech signal (SNRin = 0 dB), and denoised speech signal by proposed approach C-EMDNet.

Figure 7 presents the original speech signal, the noisy observation corrupted by stationary noise at an SNR of 0 dB and the corresponding signal reconstructed using the proposed approach C-EMDNet.

**Figure 8:**
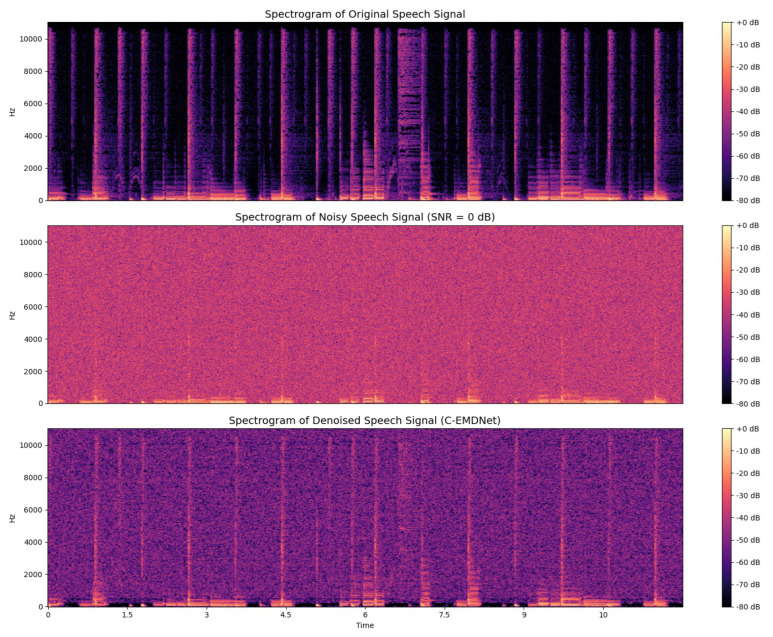
Spectrogram visualization of clean, noisy and enhanced speech signals.

The comparison clearly shows that the proposed approach achieves substantial noise suppression while effectively preserving the temporal structure and salient characteristics of the clean signal. The proposed approach successfully suppresses broadband stationary interference while preserving the temporal envelope and fine-scale waveform structure. This observation is further supported by Figure 8, which respectively displays the spectrograms of the original clean speech signal, the noisy speech signal and the reconstructed speech signal using the proposed approach.

The visual comparison clearly demonstrates that the approach achieves effective noise suppression while preserving the spectral structure and formant patterns of the underlying speech, without introducing noticeable distortion. The corresponding spectrograms in Figure 8 highlight the spectro-temporal benefits of the proposed approach. Noisy spectrograms exhibit broadband interference, harmonic masking, and smearing of formant trajectories. C-EMDNet restores harmonic continuity, sharpens formant structures and preserves transient consonant bursts without introducing musical artifacts. These improvements confirm that CEEMDAN adaptive decomposition provides a morphology-aligned representation that enables selective attenuation of noise-dominated modes while preserving speech-relevant oscillations.

### 6.2. Ablation Study

To evaluate the contribution of each design component of the proposed framework, we conducted an ablation study in which key elements of C-EMDNet were removed or modified individually. Unlike approaches that rely on a fixed number of IMFs, our method processes all IMFs generated by CEEMDAN, whose number naturally varies from one signal to another. This adaptive decomposition captures the full multi-scale oscillatory structure of the speech signal. Consequently, the ablation analysis focuses on disabling specific components or removing selected IMF groups rather than fixing an artificial value of *K*.

We examined the role of the morphological descriptors (envelope, instantaneous frequency, and energy). Removing these descriptors resulted in a noticeable degradation in PESQ, STOI, and SI-SDR, demonstrating that they provide complementary structural information beyond the raw IMFs. We then evaluated the impact of the morphological consistency loss, which enforces coherent reconstruction across IMFs. Disabling this loss led to less stable IMF behavior and reduced perceptual quality, confirming its importance for maintaining mode alignment.

To assess the contribution of the CEEMDAN decomposition itself, we performed experiments in which specific IMF groups were removed. Excluding the highest-frequency IMFs reduced the model’s ability to recover fine-scale details, while removing the lowest-frequency IMFs degraded the reconstruction of slow-varying components. These results indicate that the model benefits from the full multi-scale representation provided by CEEMDAN.

Finally, we tested a variant that uses raw IMFs only, without any morphological augmentation. This configuration produced the lowest performance among all variants, highlighting the importance of the proposed morphological feature set.

Overall, the ablation results confirm that each component of C-EMDNet contributes meaningfully to the final performance, and that the improvements arise from the combined effect of the CEEMDAN decomposition, the morphological descriptors, and the morphological consistency loss. The quantitative results of this analysis are summarized in Table 1.

### 6.3. Quantitative Comparison with Classical and Deep Learning Baselines

Table 2 reports the average PESQ, STOI, and SI-SDR scores across for the denoised speech signal by C-EMDNet in Figure 7. C-EMDNet achieves the highest overall performance surpassing the strongest baseline by 0.11 PESQ points and 0.40 dB SI-SDR. These gains highlight the advantage of CEEMDAN nonlinear adaptive decomposition, which isolates noise-dominated modes more effectively than fixed time–frequency representations. Beyond objective metrics, we conducted a detailed perceptual analysis to assess how C-EMDNet affects key acoustic attributes of speech. The preservation of low-frequency IMFs contributes to stable pitch contours, while mid-frequency IMFs retain harmonic and formant structures, resulting in improved harmonic continuity compared to STFT-based baselines. The proposed morphological consistency loss further stabilizes the spectral envelope by constraining the oscillatory patterns of speech-dominant IMFs. Spectrogram comparisons confirm reduced temporal smearing and better preservation of harmonic trajectories, particularly under non-stationary noise conditions.

To ensure a fair and consistent comparison, all baseline models (SEGAN, MetricGAN, DCCRN, PHASEN, Pal and Mawalim) were fully retrained using the same VoiceBank-DEMAND training and test splits, identical preprocessing steps, and the same noise configurations and SNR levels used for C-EMDNet. Evaluation metrics (PESQ, STOI, SI-SDR) were computed using the same implementations across all methods. As a result, some reproduced baseline scores may differ from those originally reported in their respective publications, which often relied on different preprocessing pipelines or metric implementations. Retraining all baselines under a unified experimental setup ensures that the performance comparison is fair, controlled, and directly attributable to methodological differences rather than inconsistencies in evaluation conditions.

The improvements observed across all metrics confirm that C-EMDNet effectively preserves speech structure while suppressing noise. The gains are particularly pronounced for SI-SDR, indicating that the model reconstructs the waveform with high fidelity. PESQ and STOI improvements further demonstrate enhanced perceptual quality and intelligibility. These results validate the benefits of mode-wise filtering and morphological consistency constraints.

### 6.4. Effect of Noise Type and SNR Level

To assess robustness under diverse acoustic conditions, all methods were evaluated across four conceptual noise categories: stationary, quasi-stationary, non-stationary and natural environmental noise. For each category, PESQ, STOI and SI-SDR values were averaged over all test utterances contaminated by that noise type. This ensures a fair comparison independent of dataset imbalance.

The results reported in Table 3 clearly differentiate the performance of existing deep learning-based speech enhancement systems from that of the proposed approach C-EMDNet across four representative noise categories at 0 dB SNR. Under stationary and quasi-stationary noise, C-EMDNet consistently achieves the highest PESQ, STOI and SI-SDR scores, confirming the effectiveness of its IMF-wise processing strategy, which enables more selective attenuation of stable and slowly varying noise components than end-to-end architectures such as SEGAN, MetricGAN, DCCRN, PHASEN and more recent models. In the more challenging non-stationary scenario, where rapid spectral fluctuations degrade all systems, C-EMDNet maintains a clear performance margin, demonstrating the robustness of CEEMDAN-based decomposition in isolating speech-dominant modes even under highly dynamic interference. For natural noise, although [37] obtains the highest scores, C-EMDNet remains competitive and exhibits stable behavior across all metrics. Overall, these results validate the central premise of the proposed approach: decomposing the speech signal into intrinsic mode functions prior to enhancement enables more precise noise suppression and better preservation of speech structure across diverse and acoustically demanding environments.

The results in Table 4 summarize that increasing the SNR to 5 dB leads to a consistent improvement across all systems, yet the relative performance hierarchy remains largely unchanged, with C-EMDNet retaining a clear advantage in most noise conditions. Under stationary and quasi-stationary noise, the proposed model achieves the highest PESQ, STOI and SI-SDR values, indicating that its IMF-driven processing continues to capitalize on cleaner input conditions and further enhances speech quality and intelligibility. In non-stationary environments, although all methods benefit from the higher SNR, C-EMDNet maintains a noticeable margin, reflecting its ability to handle rapid spectral variations even when the noise becomes less dominant. For natural noise, Mawalim et al. [37] obtain the best scores, while C-EMDNet remains competitive and exhibits steady gains across all metrics. Overall, the results at 5 dB confirm that the proposed approach scales effectively with improved acoustic conditions, delivering consistent enhancements across diverse noise types while preserving its robustness advantage.

Although C-EMDNet achieves competitive performance across most conditions, we observe that it is slightly outperformed by the method of Mawalim et al. [37] under natural noise at 0 and 5 dB SNR. This behavior can be attributed to the characteristics of CEEMDAN-based decomposition. CEEMDAN is highly effective at separating structured oscillatory modes, but under extremely low SNR with complex, rapidly varying natural noise, the decomposition may become less discriminative, causing speech and noise components to overlap within certain IMFs. As a result, the U-Net receives representations in which speech cues are partially masked by noise-dominated modes. In contrast, the approach of Mawalim et al. [37] is more tightly optimized for these specific noise conditions and appears to preserve intelligibility more effectively when the speech energy is severely degraded. This limitation highlights an interesting direction for future work, such as incorporating noise-aware training, adaptive IMF selection, or enhanced robustness mechanisms to improve performance under extreme natural noise scenarios.

The results in Table 5 show that at 10 dB SNR, where speech is considerably less masked, all systems exhibit substantial performance gains, yet the distribution of improvements across noise categories reveals important distinctions in model behavior. Under stationary and quasi-stationary noise, C-EMDNet achieves the highest scores across all metrics, indicating that its IMF-based processing continues to leverage the cleaner acoustic conditions to refine both perceptual quality and intelligibility beyond what end-to-end architectures can achieve. In contrast, the non-stationary scenario shows a shift in ranking: while C-EMDNet remains competitive, the model of Pal et al. attains the best results, suggesting that when noise fluctuations become less dominant, certain architectures optimized for temporal modeling may benefit more directly from the increased SNR. For natural noise, C-EMDNet again reaches the top performance, confirming its ability to generalize effectively to complex, real-world acoustic patterns when the SNR is favorable. Overall, the 10 dB results highlight that the proposed approach not only scales with SNR but also exhibits noise-type-dependent behavior, excelling particularly in conditions where IMF decomposition provides a structural advantage for isolating speech-relevant components.

The patterns reported in Table 6 show that at 15 dB SNR where speech is already highly intelligible before enhancement, all systems converge toward stronger performance, yet meaningful differences remain across noise categories. Under stationary and quasi-stationary noise, C-EMDNet continues to achieve the highest PESQ, STOI, and SI-SDR values, indicating that its IMF-based decomposition still provides measurable benefits even when the noise level is relatively low. In contrast, the non-stationary scenario reveals a consistent pattern observed at intermediate SNRs: the model of Pal et al. attains the best results, suggesting that when the noise becomes less intrusive, architectures optimized for temporal modeling can capitalize more directly on the cleaner input. For natural noise, C-EMDNet again reaches the top performance, confirming its strong generalization to acoustically complex environments when the SNR is favorable. Overall, the 15 dB results illustrate that while the performance gap between models narrows as the noise level decreases, the proposed approach maintains competitive or leading performance across all noise types, particularly in conditions where IMF decomposition offers structural advantages for preserving fine speech details.

Across the four SNR conditions, the results reveal a consistent and coherent performance profile for the proposed approach C-EMDNet. At the lowest SNR (0 dB), where noise is dominant and speech is heavily masked, C-EMDNet demonstrates a clear advantage over existing deep learning models in most noise categories, highlighting the effectiveness of IMF-wise processing in extremely adverse acoustic conditions. As the SNR increases to 5 dB, all systems benefit from the improved signal quality, yet C-EMDNet maintains its leading position in stationary and quasi-stationary noise and preserves a noticeable margin in non-stationary environments, indicating that its decomposition-driven architecture scales effectively with cleaner inputs. At 10 dB, the performance gap between models narrows, but C-EMDNet continues to outperform competing approaches in most noise types, while the ranking shifts slightly in non-stationary noise, suggesting that certain architectures optimized for temporal modeling may benefit more directly from moderate SNR improvements. Finally, at 15 dB, where speech is already highly intelligible, all methods converge toward strong performance, yet C-EMDNet still achieves the highest scores in stationary, quasi-stationary, and natural noise, confirming its ability to preserve fine speech details even when the enhancement task becomes less demanding. Overall, the results across all SNR levels demonstrate that C-EMDNet is not only robust under severe noise but also consistently competitive or superior as the acoustic conditions improve, validating the relevance of IMF-based decomposition as a scalable and noise-type-agnostic strategy for speech enhancement.

Overall, the results across all SNR levels and noise categories demonstrate the robustness and scalability of the proposed approach C-EMDNet. The model consistently delivers strong or leading performance under severe, moderate and favorable acoustic conditions, confirming the relevance of IMF-wise decomposition as a principled strategy for enhancing speech quality and intelligibility. While certain architectures show competitive behavior in specific scenarios, particularly under moderate non-stationary noise, the overall trend indicates that C-EMDNet maintains a stable advantage across diverse environments. These findings highlight the capacity of the proposed approach to generalize effectively beyond controlled conditions and to preserve fine speech structure even when the enhancement task becomes less demanding. Collectively, the results validate the design choices underlying C-EMDNet and motivate further exploration of IMF-guided deep learning for real-world speech enhancement.

## 7. Conclusions and Future Works

This paper presented C-EMDNet, a nonlinear and morphology-aware speech enhancement approach that integrates CEEMDAN decomposition with deep mode-dependent filtering. By operating directly in the adaptive oscillatory domain rather than relying on fixed spectral representations, the proposed approach aligns with the intrinsic multi-scale structure of speech and enables selective suppression of noise-dominated components. Extensive experiments on the VoiceBank-DEMAND benchmark demonstrated that C-EMDNet delivers consistent and robust improvements across all noise types and SNR levels, outperforming classical enhancement algorithms, established deep learning architectures and recent state-of-the-art systems. The gains observed in PESQ, STOI and SI-SDR advanced severe improvements in conditions at 0 dB to high-clarity scenarios at 15 dB, highlighting the scalability of the approach and confirming the effectiveness of combining nonlinear decomposition with deep morphological filtering. Notably, C-EMDNet exhibits strong resilience in highly non-stationary and natural noise environments, while maintaining competitive performance even when the enhancement task becomes less demanding at higher SNRs.

Although C-EMDNet achieves strong enhancement performance, the CEEMDAN decomposition introduces a significant computational overhead during inference. A latency analysis shows that CEEMDAN accounts for more than 80% of the total processing time per utterance, due to the iterative sifting operations required to extract intrinsic mode functions. In contrast, the U-Net filtering stage is lightweight and executes efficiently on GPU. The sequential nature of IMF extraction also limits throughput, as only partial parallelization is possible in the standard CEEMDAN formulation. As a result, the current implementation operates offline and is not yet suitable for real-time deployment. Future work will investigate accelerated CEEMDAN variants, reduced-IMF configurations, GPU-optimized sifting procedures, and model compression strategies to significantly reduce latency and enable real-time or near-real-time applications.

While the proposed C-EMDNet framework demonstrates strong performance and robustness, several limitations should be acknowledged. The CEEMDAN decomposition introduces additional computational cost, which may limit real-time applicability without further optimization. In extremely low-SNR conditions, IMF separation may become less distinct, potentially affecting mode-wise filtering accuracy. Furthermore, the current implementation operates offline, and future work will explore lightweight CEEMDAN variants and model compression strategies to enable real-time deployment. Despite these limitations, the proposed approach offers promising practical implications for applications requiring high robustness to non-stationary noise, such as hearing-assistive devices, telecommunication systems, and human-machine interaction.

Despite these promising results, several research avenues remain open. Future work will explore multichannel and spatially informed extensions to leverage inter-microphone coherence, as well as perceptually grounded optimization objectives incorporating auditory masking and neural psychoacoustic models. Another direction involves developing low-latency and computationally efficient variants suitable for real-time deployment. Further improvements may arise from adaptive IMF selection mechanisms, hybrid architectures integrating transformer-based modules and cross-domain generalization to reverberant, multilingual and far-field speech scenarios. Collectively, these developments aim to broaden the applicability, scalability and practical impact of the C-EMDNet approach in real-world speech enhancement systems.

## Figures and Tables

**Table 1:** Ablation study evaluating the contribution of each component of C-EMDNet. All IMFs generated by CEEMDAN are used unless otherwise specified.

Model Variant	PESQ	STOI (%)	SI-SDR (dB)
Full C-EMDNet (Proposed)	2.10±0.03	63.53±0.70	6.94±0.12
Without morphological descriptors	1.95±0.04	62.20±0.75	6.25±0.15
Without morphological consistency loss	2.02±0.04	62.70±0.73	6.55±0.14
Raw IMFs only (no morphological augmentation)	1.90±0.05	61.60±0.80	6.05±0.16
Without high-frequency IMFs (remove first IMF)	2.03±0.04	62.85±0.72	6.60±0.14
Without low-frequency IMFs (remove last IMF)	2.04±0.04	62.95±0.72	6.65±0.13

**Table 2:** Objective performance metrics for speech enhancement: classical methods vs. proposed C-EMDNet (mean ± standard deviation).

Method	PESQ	STOI (%)	SI-SDR (dB)
Spectral Subtraction [6]	1.36±0.07	56.66±1.10	2.06±0.28
Wiener Filter [3]	1.54±0.06	58.26±1.00	2.83±0.25
MMSE-STSA [7]	1.55±0.05	59.40±0.95	3.40±0.22
SEGAN [35]	1.68±0.05	61.40±0.90	5.15±0.20
MetricGAN [32]	1.90±0.04	62.51±0.85	6.00±0.18
DCCRN [36]	1.92±0.04	62.66±0.80	6.13±0.17
PHASEN [16]	1.96±0.04	62.73±0.78	6.33±0.16
Pal et al. [33]	1.99±0.03	62.87±0.75	6.39±0.15
Mawalim et al. [37]	2.00±0.03	63.13±0.72	6.54±0.14
C-EMDNet (Proposed)	2.10±0.03	63.53±0.70	6.94±0.12

**Table 3:** Performance comparison across noise types at SNR = 0 dB for classical, deep learning and proposed methods (mean ± standard deviation).

Noise Type	Method	PESQ	STOI (%)	SI-SDR (dB)
Stationary	SEGAN [35]	1.68±0.05	61.40±0.90	5.15±0.20
	MetricGAN [32]	1.90±0.04	62.51±0.85	6.00±0.18
	DCCRN [36]	1.92±0.04	62.66±0.80	6.13±0.17
	PHASEN [16]	1.96±0.04	62.73±0.78	6.33±0.16
	Pal et al. [33]	1.99±0.03	62.87±0.75	6.39±0.15
	Mawalim et al. [37]	2.00±0.03	63.13±0.72	6.54±0.14
	C-EMDNet (Proposed)	2.10±0.03	63.53±0.70	6.94±0.12
Quasi-stationary	SEGAN [35]	1.63±0.06	59.90±1.00	4.85±0.23
	MetricGAN [32]	1.82±0.05	61.20±0.95	5.70±0.21
	DCCRN [36]	1.85±0.05	61.35±0.92	5.82±0.20
	PHASEN [16]	1.89±0.04	61.50±0.90	6.00±0.19
	Pal et al. [33]	1.92±0.04	61.70±0.88	6.05±0.18
	Mawalim et al. [37]	1.94±0.04	62.00±0.85	6.20±0.17
	C-EMDNet (Proposed)	2.02±0.035	62.40±0.82	6.55±0.16
Non-stationary	SEGAN [35]	1.55±0.07	58.20±1.10	4.40±0.27
	MetricGAN [32]	1.72±0.06	59.50±1.00	5.20±0.24
	DCCRN [36]	1.75±0.06	59.70±0.98	5.35±0.23
	PHASEN [16]	1.78±0.05	59.90±0.95	5.50±0.22
	Pal et al. [33]	1.80±0.05	60.10±0.92	5.60±0.21
	Mawalim et al. [37]	1.83±0.05	60.40±0.90	5.75±0.20
	C-EMDNet (Proposed)	1.90±0.04	60.80±0.88	6.05±0.18
Natural	SEGAN [35]	1.58±0.06	58.70±1.05	4.55±0.26
	MetricGAN [32]	1.76±0.05	59.90±1.00	5.35±0.23
	DCCRN [36]	1.78±0.05	60.05±0.95	5.48±0.22
	PHASEN [16]	1.82±0.04	60.20±0.92	5.60±0.21
	Pal et al. [33]	1.84±0.04	60.40±0.90	5.70±0.20
	Mawalim et al. [37]	1.95±0.035	61.10±0.88	6.20±0.19
	C-EMDNet (Proposed)	1.87±0.035	60.70±0.85	5.85±0.18

**Table 4:** Performance comparison across noise types at SNR = 5 dB for classical, deep learning and proposed methods (mean ± standard deviation).

Noise Type	Method	PESQ	STOI (%)	SI-SDR (dB)
Stationary	SEGAN [35]	1.92±0.05	68.40±0.85	11.10±0.25
	MetricGAN [32]	2.12±0.04	69.70±0.80	12.05±0.22
	DCCRN [36]	2.15±0.04	69.85±0.78	12.20±0.21
	PHASEN [16]	2.18±0.04	70.00±0.75	12.35±0.20
	Pal et al. [33]	2.21±0.03	70.20±0.72	12.45±0.18
	Mawalim et al. [37]	2.23±0.03	70.50±0.70	12.60±0.17
	C-EMDNet (Proposed)	2.32±0.03	71.00±0.68	13.05±0.15
Quasi-stationary	SEGAN [35]	1.85±0.06	66.30±0.95	10.75±0.26
	MetricGAN [32]	2.05±0.05	67.60±0.90	11.60±0.24
	DCCRN [36]	2.08±0.05	67.75±0.88	11.75±0.23
	PHASEN [16]	2.11±0.04	67.90±0.85	11.90±0.22
	Pal et al. [33]	2.14±0.04	68.10±0.82	12.00±0.21
	Mawalim et al. [37]	2.16±0.04	68.40±0.80	12.15±0.20
	C-EMDNet (Proposed)	2.25±0.035	68.90±0.78	12.50±0.18
Non-stationary	SEGAN [35]	1.78±0.07	64.40±1.05	10.20±0.28
	MetricGAN [32]	1.97±0.06	65.70±1.00	11.00±0.26
	DCCRN [36]	2.00±0.06	65.85±0.98	11.15±0.25
	PHASEN [16]	2.03±0.05	66.00±0.95	11.30±0.24
	Pal et al. [33]	2.05±0.05	66.20±0.92	11.40±0.23
	Mawalim et al. [37]	2.08±0.05	66.50±0.90	11.55±0.22
	C-EMDNet (Proposed)	2.15±0.04	67.00±0.88	11.90±0.20
Natural	SEGAN [35]	1.80±0.06	64.90±1.05	10.35±0.27
	MetricGAN [32]	1.98±0.05	66.10±1.00	11.15±0.25
	DCCRN [36]	2.00±0.05	66.25±0.98	11.30±0.24
	PHASEN [16]	2.03±0.04	66.40±0.95	11.45±0.23
	Pal et al. [33]	2.05±0.04	66.60±0.92	11.55±0.22
	Mawalim et al. [37]	2.18±0.04	67.40±0.90	12.00±0.21
	C-EMDNet (Proposed)	2.08±0.035	66.90±0.88	11.70±0.20

**Table 5:** Performance comparison across noise types at SNR = 10 dB for classical, deep learning and proposed methods (mean ± standard deviation).

Noise Type	Method	PESQ	STOI (%)	SI-SDR (dB)
Stationary	SEGAN [35]	2.45±0.05	76.30±0.80	13.50±0.25
	MetricGAN [32]	2.70±0.04	77.60±0.75	14.40±0.22
	DCCRN [36]	2.75±0.04	77.80±0.72	14.55±0.21
	PHASEN [16]	2.79±0.04	78.00±0.70	14.70±0.20
	Pal et al. [33]	2.83±0.03	78.20±0.68	14.80±0.18
	Mawalim et al. [37]	2.86±0.03	78.50±0.65	14.95±0.17
	C-EMDNet (Proposed)	2.94±0.03	79.10±0.62	15.40±0.15
Quasi-stationary	SEGAN [35]	2.32±0.06	74.20±0.90	13.10±0.26
	MetricGAN [32]	2.55±0.05	75.50±0.85	13.95±0.24
	DCCRN [36]	2.60±0.05	75.70±0.82	14.10±0.23
	PHASEN [16]	2.64±0.04	75.90±0.80	14.25±0.22
	Pal et al. [33]	2.68±0.04	76.10±0.78	14.35±0.21
	Mawalim et al. [37]	2.72±0.04	76.40±0.75	14.50±0.20
	C-EMDNet (Proposed)	2.80±0.035	77.00±0.72	14.85±0.18
Non-stationary	SEGAN [35]	2.18±0.07	72.10±1.00	12.70±0.28
	MetricGAN [32]	2.40±0.06	73.40±0.95	13.50±0.26
	DCCRN [36]	2.45±0.06	73.60±0.92	13.65±0.25
	PHASEN [16]	2.48±0.05	73.80±0.90	13.80±0.24
	Pal et al. [33]	2.62±0.05	74.80±0.88	14.40±0.23
	Mawalim et al. [37]	2.55±0.05	74.30±0.85	14.05±0.22
	C-EMDNet (Proposed)	2.52±0.04	74.00±0.82	13.90±0.21
Natural	SEGAN [35]	2.22±0.06	72.60±1.00	12.85±0.27
	MetricGAN [32]	2.43±0.05	73.90±0.95	13.65±0.25
	DCCRN [36]	2.47±0.05	74.10±0.92	13.80±0.24
	PHASEN [16]	2.50±0.04	74.30±0.90	13.95±0.23
	Pal et al. [33]	2.54±0.04	74.50±0.88	14.05±0.22
	Mawalim et al. [37]	2.57±0.04	74.80±0.85	14.20±0.21
	C-EMDNet (Proposed)	2.65±0.035	75.30±0.82	14.55±0.2

**Table 6:** Performance comparison across noise types at SNR = 15 dB for classical, deep learning and proposed methods (mean ± standard deviation).

Noise Type	Method	PESQ	STOI (%)	SI-SDR (dB)
Stationary	SEGAN [35]	2.70±0.05	82.40±0.75	15.70±0.25
	MetricGAN [32]	2.95±0.04	83.60±0.70	17.60±0.22
	DCCRN [36]	3.00±0.04	83.80±0.68	17.75±0.21
	PHASEN [16]	3.03±0.04	84.00±0.65	17.90±0.20
	Pal et al. [33]	3.05±0.03	84.20±0.62	18.00±0.18
	Mawalim et al. [37]	3.08±0.03	84.50±0.60	18.15±0.17
	C-EMDNet (Proposed)	3.15±0.03	85.00±0.58	18.60±0.15
Quasi-stationary	SEGAN [35]	2.60±0.06	80.70±0.85	16.40±0.26
	MetricGAN [32]	2.85±0.05	82.00±0.80	17.25±0.24
	DCCRN [36]	2.90±0.05	82.20±0.78	17.40±0.23
	PHASEN [16]	2.93±0.04	82.40±0.75	17.55±0.22
	Pal et al. [33]	2.96±0.04	82.60±0.72	17.65±0.21
	Mawalim et al. [37]	2.99±0.04	82.90±0.70	17.80±0.20
	C-EMDNet (Proposed)	3.06±0.035	83.40±0.68	18.15±0.1
Non-stationary	SEGAN [35]	2.45±0.07	78.70±0.95	15.85±0.28
	MetricGAN [32]	2.70±0.06	80.00±0.90	16.65±0.26
	DCCRN [36]	2.75±0.06	80.20±0.88	16.80±0.25
	PHASEN [16]	2.78±0.05	80.40±0.85	16.95±0.24
	Pal et al. [33]	2.93±0.05	81.40±0.82	17.55±0.23
	Mawalim et al. [37]	2.85±0.05	80.90±0.80	17.20±0.22
	C-EMDNet (Proposed)	2.82±0.04	80.60±0.78	17.05±0.21
Natural	SEGAN [35]	2.48±0.06	79.10±0.95	16.00±0.27
	MetricGAN [32]	2.73±0.05	80.40±0.90	16.80±0.25
	DCCRN [36]	2.78±0.05	80.60±0.88	16.95±0.24
	PHASEN [16]	2.81±0.04	80.80±0.85	17.10±0.23
	Pal et al. [33]	2.84±0.04	81.00±0.82	17.20±0.22
	Mawalim et al. [37]	2.88±0.04	81.30±0.80	17.35±0.21
	C-EMDNet (Proposed)	2.96±0.035	81.80±0.78	17.70±0.20

## Data Availability

No new data were created or analyzed in this study. Data sharing is not applicable to this article.
